# Amalgamation of St. George's and Westminster Hospitals

**Published:** 1913-07-19

**Authors:** 


### AMALGAMATION OF ST. GEORGE'S AND WESTMINSTER HOSPITALS.

### Unanimous Approval by the St. George's Governors.

There was a very full attendance at the Special Court of Governors of St. George's Hospital held on Thursday, July 10, to consider the question of the amalgamation of these two hospitals, and the building of one large hospital in South-West London. The chair was taken by Mr. A. William West, treasurer of the hospital, who conveyed to the meeting Lord Plymouth's regret at his inability to be present. Among the governors and staff present were Viscount Maidstone, the Right Hon. Sir William Carington, the Hon. James Mansfield, Sir Thomas Dewar, the Hon. Mrs. William West, Sir Henry Burdett, Colonel Wilson, Colonel Skinner, Colonel Conway Bishop, the Right Hon. Lawrence Hardy, M.P., Captain Claude Hay, Mr. Warrington Haward (consulting surgeon), Mr. Richard Thorp, Dr. Latham, Dr. Tuke, Dr. E. I. Spriggs, Mr. Adams Frost, Mr. Jaffrey, Dr. Wilfred Fox, and Mr. G. E. Friend (medical superintendent).

The Chairman read the following telegram which had been received from the Duke of Grafton: "I trust that no decision will be made until a special meeting of all subscribers has been called. Sorry unable to attend myself." He added that evidently his Grace did not understand that the present Court was convened specially for that purpose. The business was the consideration of the proposed amalgamation of the two hospitals and the removal to a new hospital to be built. He reminded the meeting that the last date on which a Special Court was summoned to deal with the matter was June 1906, when removal was negatived owing to no adequate offer having been made for a suitable site. But he would be able to show that the circumstances had now altered, as previously there was no question of amalgamation with another hospital of high repute and uniting resources with removal to a new district. Moreover, the House Committee had now received the following offer, which he thought must be regarded as an adequate one:—

"Subject to a formal contract and to my being able to come to a satisfactory arrangement with the Duke of Westminster for his rights of pre-emption over a certain portion of the site, I hereby offer to purchase the land upon which St. George's Hospital now stands, together with the buildings thereon and the houses numbered 1, 3, 5, 7 and 9 Knightsbridge, belonging to the hospital, containing on the whole 1 acre, 2 roods, 33 poles, and 13½ yards or thereabouts, for the sum of £460,000, payable as to £285,000 in cash, and £175,000 to be secured by a second mortgage at 4½ per cent. per annum over the whole of the site. No greater prior mortgage than £350,000 to be put upon the property. A deposit of £10,000 to be paid upon the signing of the contract, and the hospital to have the right to retain such internal fixtures, fittings, floors, and stained-glass as you may desire."

It would, he said, have been more satisfactory if the offer had been on a cash basis, but assuming the site was worth the amount being offered for it, together with the sum to be paid to the Duke of Westminster's estate in two years' time, then the £175,000 second mortgage should be secure on the site alone. But even if this were not so, there was still the personal security of the proposer, whose intention was to spend at least £600,000 upon the new hotel which it was intended to erect on the site. The offer was a net one to the hospital, as it would not have to pay the agent's commission. On June 11 St. George's House Committee passed the following resolution:—

"Without committing itself to an opinion as to the details of the scheme, the Committee approves the proposal to amalgamate the hospitals and removal to another site, and proposes that a joint sub-committee be appointed to prepare a detailed scheme and report for submission to the Court of Governors."

The joint sub-committee of this and the Westminster Hospitals had held several meetings, at which the problems involved had been carefully considered; and they were unanimous in the view that amalgamation and removal of the two hospitals to another site as one joint hospital was desirable. Assuming that the proposal would prove acceptable to the Board, two sites had been entertained: one at Clapham Common, the other at Wandsworth Bridge, the latter some 8 or 9 acres in extent, with a frontage to the river. The joint sub-committee reported that the balance of advantage was with the Wandsworth site. Proceeding, Mr. West said that, in his view, the great argument in favour of removal was that St. George's was now no longer in direct touch with the poor as it used to be, as the character of the district had changed. Removal to Wandsworth would establish a great hospital within a stone's-throw of the district from which most of St. George's patients came. The gradual outward squeezing to the further west and south-west of Hyde Park Corner of the poorer population was well evidenced by the following figures:—

In 1891, 451 in-patients were admitted from Fulham and Walham Green districts, whereas in 1910 the number was 732. In 1891, 567 in-patients were received from Pimlico, but in 1910 this district had contributed only 330. The extra journey implied by these figures meant a great hardship on the very poor, owing to the 'bus fares to and fro.

Moreover, supposing the sale of the two sites to be effected, if the invested funds and property of the two institutions are brought into a common account, then after purchasing a new site of 8 acres and building a new hospital of 500 beds there would remain as an endowment fund a sum of between £850,000 and £900,000. It was important to point out that the hospital was not asked to relinquish the present site for two years, by which time the new building should be ready, and hence the work of the hospital need not suffer any material interruption. In order that the district should not be left entirely bereft of hospital attention, a proposal was being entertained to establish a centre for first-aid treatment, to which cases of accident in the neighbourhood could be taken, and, if necessary, they could be removed to the new hospital by motor ambulance. The hospital already possessed land in Montpelier Street available for the purpose. It must, also, be more economical to work one hospital of 500 beds than two independent institutions with a similar combined accommodation.

A further consideration was the advantage which would accrue to the medical school. The way to secure the best treatment for the patients was to give every possible advantage to the medical school: a good hospital and a good school went together. Under the present scheme there would not only be new laboratories, but the amalgamation of the two schools would provide a larger number of entries, and consequently a wider selection for house officers, etc. Further, the Board of Education desired to concentrate their help on the larger and more important schools; and the Royal Commission on University Education in London had reported strongly in favour of hospitals of 500 to 600 beds as being worthy of their support. The spirit in which the Westminster Hospital Committee had approached the question seemed to promise that there would be no insuperable obstacles. The Duke of Westminster's estate had an interest in the present St. George's site, but his Grace's Board had met the hospital in a most reasonable way, and had it not been for the desire of that Board to benefit the hospital as much as they could such a favourable proposal could not have been now submitted.

The resolutions which he intended to propose * would, if carried, enable a start to be made as soon as the necessary Act of Parliament to enable the two hospitals to amalgamate had been granted and passed. The proposals had been laid before the Charity Commissioners, who, so far as they could see at present, would raise no objections; indeed, they had acted most kindly and helpfully throughout, and were anxious to assist in obtaining the necessary Act of Parliament.

In conclusion, the CHAIRMAN declared his willingness to answer any questions.

Mr. KEYSER said it afforded him pleasure in seconding the resolution. That he had been asked to do so was due to the fact that he was the author of the resolution which was carried by the House Committee that the matter should be laid before the Special Court. He felt there could be no question that in removing the hospital as suggested the best use was being made of the trust imposed upon the Court in administering that great charity. The power for good would be enormously enhanced, because the hospital would be much more accessible to the poor who needed it most, and while a much larger institution than the present one would be provided, it would be worked on a much more economical basis. He did not see, therefore, how the Court could refuse to sanction the scheme. Any objection must be a sentimental one, and sentiment, of course, weighed with all, especially with those who had been longest connected with the hospital. He had himself been in regular attendance for twenty years, and it would cause him a pang to see any other building erected on the site; but compared with the considerations of much greater usefulness and power, such feelings could not be allowed to enter into the argument. A great point was the prospect of a more efficient and complete medical school, for he was emphatically of the opinion that the interests of the medical school and of the hospital could not be separated. (Applause.) And if the relative indebtedness could be measured in pounds, shillings, and pence, it would be found that the hospital was the debtor to the school, rather than the reverse; the gratuitous services rendered to the hospitals by those attached to the medical schools represented an enormous sum, which was never taken into account. The scheme would result in benefit in every way, and he hoped it would be carried.

Mr. WARRINGTON HAWARD (consulting surgeon to the hospital) confessed, as one who had worked within those walls for over half a century, that he felt a pang at the thought of removal of the hospital from Hyde Park Corner. But sentiment should be allowed no part in this decision; the consideration should be the welfare of the poor, for whose benefit the hospital was founded. It would be a great achievement to build a large hospital, with all modern methods and facilities, in the midst of the poor population whom it served. When he was a student, there was a slum at the back of Sloane Street, and many midwifery cases were attended there; but it was now one of London's fashionable localities. Similarly with regard to the neighbourhood of Victoria Station the poor had been driven further and further away. The expenditure at St. George's at present was greatly affected by its restricted site; hence there was not room for any enlargements which might be dictated by the desire to keep pace with the advancements in medicine and surgery. The nursing staff were compelled to reside at some distance from the hospital. And the administrative staff for a hospital of 500 beds would probably be little, if any, greater than for St. George's alone. There need be no sentimental objection against St. George's joining with Westminster, as the two hospitals were originally one, so it would be merely a reversion to an earlier arrangement. The staffs of the two hospitals were much interested in the matter, and it was a pleasure to him to know of the amicable feelings entertained by the medical men attached to both hospitals. He hoped personal considerations would be relegated to the background, and that the resolutions would be carried.

Mr. R. H. C. HARRISON (a member of the House Committee) said his chief reason for supporting the scheme was a financial one, and the second was the greatly increased accessibility of the hospital to the poor. The hospital was occupying a site now worth £20,000 a year, and it gave no proportional advantage, but £12,000 to £14,000 a year had to be found in order to meet the expenditure. Having served on the joint committee, he was much impressed by the advantages which the scheme offered, whether the site chosen be Clapham or Wandsworth. It had been thought by some that people of means who drove or rode in the Park would be moved by the sight of the hospital to contribute to its funds; but that had not been the experience. He strongly supported the proposal.

Mr. G. R. TURNER (senior surgeon) said he would like to speak in two capacities, and first as one who formerly was much against the removal of the hospital from its present position. He did not think those who had changed their view need stand in any penitential sheet; years ago, as circumstances were, he thought the course pursued was the right one. Matters had now, however, assumed a different complexion. Not only had the neighbourhood of the hospital altered, but the medical school had tried the experiment of being a clinical school without teaching the elementary subjects. Though an exceedingly good class of student came, the number was not so great as it used to be when all the subjects of medical education were included. He hoped that with the carrying out of the present scheme the school would rank with that of St. Bartholomew's, and carry on the complete education. In the past they found that some of their students who went to learn the elementary subjects at King's College and University had drifted to those colleges for their clinical courses also, instead of returning to St. George's. In consequence, there had sometimes been a shortage in the hospital not only of dressers in the wards, but of men to fill such posts as house physician and house surgeon. That reacted badly in every way, not only on the staff—who were not spurred by any incentive to teach when there was scarcely anybody to teach—but on the sick

* These resolutions were published in THE HOSPITAL of July 12, p. 457.

poor also, because the gulf which separated the nurse—however well taught—from the educated medical student was very great. The possibility of reverting to a full course and to an enlarged sphere provided an additional incentive to the adoption of the new proposals. All would agree with Mr. Harrison's contention that the occupation of the Hyde Park Corner site was an expensive luxury, in which their experience showed the hospital could not longer indulge.

Mr. Richard Thorp (a member of the House Committee) said he was glad to learn of the conversion of the medical members to the idea of removal, which they formerly opposed, though he believed on the former date there was an even better case made out than now, for it would have retained present patients and catered better for Fulham and Walham Green. He hoped the new hospital would not be restricted to 500 beds, because St. George's had at present 360, and Westminster had 220, a total of 580. He still hoped it might be possible to get a site in the neighbourhood which was mentioned when the matter came up before. He would have liked a second mortgage to be avoided, as second mortgages had a habit of not being a mortgage at all. He would support the scheme with a full heart if he could feel assured that the poor of Fulham and Walham Green were not being overlooked, for the Wandsworth site was off the routes of traffic, and patients from these districts would be compelled to walk at least a mile and a half to get to a hospital on this site.

The Chairman pointed out that the hospital was not committed to the Wandsworth site at all: if another site more suitable could be found in another South-Western locality they would fall in with it. But it would have been no use to suggest to the Court the removal of the hospital unless a site, or, in fact, two sites, could also have been pointed to. And the resolution stated *at least* 500 beds.

Dr. E. M. Wilson laid stress on the restitution, under this scheme, of a full medical course. It was scarcely to be expected that men would come back to St. George's for clinical training only. He would have liked to hear what money would be available for the erection of the new schools and laboratories. It seemed rather a large order to give to the House Committee, by the third resolution, *carte blanche* to make all necessary arrangements.

The Chairman pointed out that the third resolution applied only to the Duke of Westminster's estate. In reply to further questions, he said the expenditure of the two hospitals now was £60,000 a year. He hoped that those who had been subscribing and giving donations to the hospital would continue to do so. People residing on the north side of Hyde Park mostly went to St. Mary's, Paddington.

The resolution was carried *nem. con.*

